# Effect of BG-Lures on the Male *Aedes* (Diptera: Culicidae) Sound Trap Capture Rates

**DOI:** 10.1093/jme/tjab121

**Published:** 2021-07-08

**Authors:** Kyran M Staunton, Joelyn Goi, Michael Townsend, Scott A Ritchie, Jacob E Crawford, Nigel Snoad, Stephan Karl, Thomas R Burkot

**Affiliations:** 1 College of Public Health, Medical and Veterinary Sciences, James Cook University, Smithfield, Australia; 2 Australian Institute of Tropical Health and Medicine, James Cook University, Smithfield, Australia; 3 Vector-Borne Diseases Unit, PNG Institute of Medical Research, Madang, 511 Madang Province, Papua New Guinea; 4 Debug, Verily Life Sciences, South San Francisco, CA, USA

**Keywords:** *Aedes aegypti*, *Aedes albopictus*, mosquito trap, dengue, sound lure

## Abstract

With global expansion of the two main vectors of dengue, *Aedes aegypti* (Linnaeus, Diptera: Culicidae) and *Aedes albopictus* (Skuse, Diptera: Culicidae), there is a need to further develop cost-effective and user-friendly surveillance tools to monitor the population dynamics of these species. The abundance of *Ae. aegypti* and *Ae. Albopictus,* and associated bycatch captured by Male *Aedes* Sound Traps (MASTs) and BG-Sentinel (BGS) traps that were unbaited or baited with BG-Lures were compared in Cairns, Australia and Madang, Papua New Guinea. Mean male *Ae. aegypti* and *Ae. albopictus* catch rates in MASTs did not significantly differ when deployed with BG-Lures. Similarly, males of both these species were not sampled at statistically different rates in BGS traps with or without BG-Lures. However, MASTs with BG-Lures caught significantly less male *Ae. aegypti* than BGS traps baited with BG-Lures in Cairns, and MASTs without BG-Lures caught significantly more male *Ae. albopictus* than BGS traps without BG-Lures in Madang. Additionally, BG-Lures significantly increased female *Ae. aegypti* catch rates in BGS traps in Cairns. Lastly, bycatch capture rates in BGS traps were not significantly influenced by the addition of the BG-Lures. While this study provides useful information regarding the surveillance of *Ae. aegypti* and *Ae. albopictus* in these locations, further development and investigation is required to successfully integrate an olfactory lure into the MAST system.

Surveillance of *Aedes aegypti* and *Aedes albopictus* (Skuse, Diptera: Culicidae) is crucial to detect incursions of these species as they continue to expand their global distributions (Kraemer et al. 2015, Kraemer et al. 2019) as well as to assess disease risk and to evaluate the efficacy of vector control interventions (Akaratovic et al. 2017). The BG-Sentinel (BGS) trap (Biogents, Regensburg, Germany) is the current gold-standard mosquito trap for *Ae. aegypti* and *Ae. albopictus* adult surveillance. While highly effective, the BGS trap unfortunately frequently captures large numbers of a variety of non-target arthropods (bycatch), requiring substantial staff time to sort through to find the targeted mosquitoes, and requires mains power or 12V batteries to operate (Azil et al. 2015).

Mosquitoes are attracted to BGS traps by the contrasting dark and white colours of the trap as well as by chemical attractants, such as carbon dioxide (Pombi et al. 2014, Roiz et al. 2016, Wilke et al. 2019) or human skin scent mimics (Hoel et al. 2007, Akaratovic et al. 2017, Degener et al. 2019) such as the BG-Lure (Biogents, Regensburg, Germany; consisting of ammonia, lactic acid, and caproic acid). The BG-Lure increases mosquito flight activity around the trap but does not induce landing (Martin Geier, personal communication, Biogents). Male *Aedes* mosquitoes have been well documented to swarm around and mate near hosts (Oliva et al. 2014). While carbon dioxide is well established as an effective mosquito attractant (Pombi et al. 2014, Roiz et al. 2016, Wilke et al. 2019), the attractiveness of human skin scent mimics is less consistent. Catch rates of both *Ae. aegypti* and *Ae. albopictus* were increased in BGS traps with human skin scent mimics in most (Barrera et al. 2013, Pombi et al. 2014, Arimoto et al. 2015, Amos et al. 2020, Visser et al. 2020), but not all studies (Williams et al. 2006, Roiz et al. 2016) with the strain of mosquito potentially influencing responses to chemical lures (Williams et al. 2006).

Most mosquito traps, including the BGS trap, were designed to capture the female mosquitoes, which bite and transmit pathogens. However, monitoring male mosquitoes can also indicate seasonal trends and distributions (Akaratovic et al. 2017). Recently, mosquito control strategies using the mass release of male mosquitoes have raised the interest in male surveillance (Crawford et al. 2020). Therefore, there has recently been renewed interest in targeting male *Aedes* through the development of traps using acoustic lures that mimic female wingbeat frequencies to attract males (Stone et al. 2013, Johnson and Ritchie 2016, Balestrino et al. 2016, Jakhete et al. 2017).

The Male *Aedes* Sound Trap (MAST) uses a sound lure to attract and capture male *Ae. aegypti* and *Ae. albopictus* into a clear capture container (Swan et al. 2020, Staunton et al. 2021a). Males entering the capture container are retained by being either being killed by an insecticide (MAST Spray) or, for deployment in locations with insecticide resistance, captured on a sticky panel (MAST Sticky; Staunton et al. 2021a). The MAST trap and BGS trap (version 2) without any lure caught comparable numbers of male *Ae. aegypti* and/or *Ae. Albopictus* in the Pacific region and the Americas, but with significantly less bycatch in the MAST (Staunton et al. 2021a, Staunton et al. 2021b).

Here, the effectiveness of MASTs and BGS traps, with and without human skin scent mimics (BG-Lures), were compared for catches of *Ae. aegypti* in Cairns, Australia and *Ae. albopictus* in Madang, Papua New Guinea. We hypothesized that the BG-Lure would increase the likelihood of male *Aedes* to fly near the MAST entrance and thereby increase MAST entry in response to the sound lure.

## Methods

### Semi-Field Trials

A series of trials between 18 September and 14 November 2019 was conducted with 800 adult *Ae. aegypti* mosquitoes (50:50 male to female ratio, 4–5 d old post-eclosion) in a large semi-field cage (Ritchie et al. 2011) in the James Cook University Mosquito Research Facility in Cairns, Australia. Mosquitoes tested were F5 (maintained in controlled-temperature rooms at 28°C and 70% RH), having been field-collected from throughout Cairns 10 mo prior. One hour after mosquito release, two traps were installed 5 m apart, facing each other, inside the cage. After 20 min traps were removed and captured mosquitoes were knocked down using CO_2_ and counted by sex. Traps were reset with location randomly assigned between the same two locations for each trial until 21–24 trials were performed.

The influence of the location of the BG-Lure was evaluated by 3 experiments comparing numbers of *Ae. aegypti* captured to MAST with BG-Lure positioned (1) externally and on top of the MAST-Spray capture chamber (where the lure was considered to be most exposed to the surrounding air and therefore potentially permeate furthest and therefore be most effective; Supp Fig. S1A [online only]), (2) inside the MAST Spray capture chamber (Supp Fig. S1B [online only]) and (3) inside the MAST Sticky capture chamber. In each experiment, the numbers of *Ae. aegy*pti captured by a MAST with BG-lure were compared to a MAST without BG-Lure. Detailed descriptions of the MAST-Spray and MAST-Sticky traps are available from Staunton et al. (2021a).

Sound lures were set to 550 Hz, 60 dB at trap entrance with an intermittent tone (30 s on-off) these settings were consistent with work demonstrating effective capture rates and lower bycatch, relative to unbaited BGS traps (Staunton et al. 2021b). A commercial residual insecticide (Mortein surface spray, Reckitt Benckiser, West Ryde, NSW, Australia) was applied to the MAST Spray container’s internal surface 24 h prior to trials.

### Field Trials

Field trials of four different trap types in a Latin square design with two complete rotations occurred between 8 January–17 March 2020 in Cairns and 7 February–12 May 2020 in Madang. In Cairns, the *Ae. aegypti* are infected with the *w*Mel strain of *Wolbachia* (Ryan et al. 2019). We compared four traps: BGS traps version 2 with and without BG-Lure and the MAST Spray with or without BG-Lures inside their capture container. Mortein surface spray (Reckitt Benckiser, West Ryde, NSW, Australia) was applied to the MAST Spray capture container’s internal surface 24 h prior to starting each Latin square. Sound lures were operated at 550 Hz intermittently (30 s on-off) and at 60 dB at the trap entrance.

In Madang the MAST Sticky was used due to local insecticide resistance (Demok et al. 2019). Yellow sticky panels (Trappit, manufactured by Entosol (Australia) Pty Ltd, Roselands), 50 × 70 mm in size, were placed within these MAST Sticky versions and replaced weekly. The BG-Lure was placed horizontally inside the killing chamber of the MAST Sticky version (Supp Fig. S1B [online only]). In Cairns, BGS traps were operated using mains power while in Madang the BGS traps were powered by car batteries (12 V, 50 Ah; manufactured by Bolt (Guangdong, China) or Yuasa (Kyoto, Japan)). Madang BGS traps were serviced on days 4 and 7 of each week to replace batteries. MAST traps were serviced weekly, by ensuring that the sound lures were operational, removing caught specimens and, for the MAST Sticky, replacing the sticky panel. All traps were randomly rotated each week through the Latin square design.

### Data Analysis

All analyses were performed using RStudio in the R statistical environment ver. 3.5.3 (R Core Team 2017). For semi-field cage trials, we fit treatment (trap type) as well as ‘trap location’ (to account for inconsistent mosquito densities throughout the cage) to the response variable of male *Ae. aegypti* abundance caught per trap from each trial. We used a generalized linear model with a Poisson distribution and log link function with ‘glmer’ in the *lme4* package (Bates et al. 2015). Interactions between fixed factors were not significant so were removed to generate the least complex adequate model. Catches varied throughout the day due to natural variations in activity and the removal of captured males from the total cage population. Therefore, we included an offset in the model, comprised of the total number of males caught by both traps during each trial, to account for such variation between trials. Lastly, we analyzed the effect of predictors with an analysis of deviance using the ‘Anova’ function and *car* package (Fox and Weisberg 2011).

For field data sets, we fit treatment (trap type) to response variable count data with a generalized linear mixed model (GLMM) with negative bionomial distributions (initial runs with Poisson distributions were consistently overdispersed) and log link functions using ‘glmer.nb’ in the *lme4* package (Bates et al. 2015). Influences on response data from location and time were accounted for by incorporating ‘trap location’ and ‘week’ as random factors in the model. Data from three BGS traps in PNG Latin squares, which did not operate properly for the entire week, were not included in the data sets and an offset parameter was included in the model to account for BGS trap fails which specified the number of days (out of seven) each trap was operational. This offset parameter was not included in the Cairns models as there were no trap failures. We then analyzed the effect of predictors with an analysis of deviance using the ‘Anova’ function within the *car* package (Fox and Weisberg 2011). We used post-hoc Tukey comparisons to determine significant differences among the least-squares means of treatment groups using the ‘emmeans’ function within the *emmeans* package (Lenth et al. 2019). While taxa of interest were also analyzed using the above technique from BGS bycatch, these investigations were not repeated for MAST bycatch due to very low counts within each group.

## Results

### Semi-Field Trials

#### BG-Lure on Top of MAST Capture Chamber

**Fig. 1.**
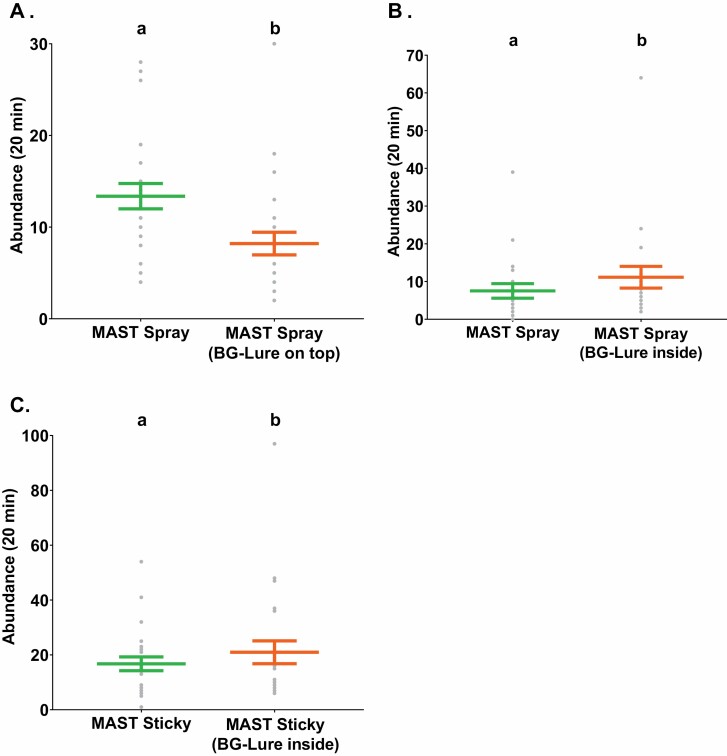
Mean abundance of male *Ae. aegypti* in the Semi-field cage for comparisons between (A) the unbaited MAST Spray and the MAST Spray with the BG-Lure on top of the MAST capture chamber (*n* = 24), (B) the unbaited MAST Spray and the MAST Spray with the BG-Lure inside the MAST capture chamber (*n* = 21) and (C) the unbaited MAST Sticky and the MAST Sticky with the BG-Lure inside the MAST capture chamber (*n* = 24). Different letters above points indicate significantly different groups (analysis of deviance, *P* < 0.05).

There was a significant decrease (*χ*^2^ = 32.1, *df* = 1, *P* < 0.05, *n* = 24) in the mean abundances of male *Ae. aegypti* caught by the MAST Spray traps when the BG-Lure was on top of the capture chamber (8. 2 ± 1.2; mean ± S. E.) compared to MAST Spray traps without the BG-Lure (13. 4 ± 1.4; Fig. 1A). Trap location significantly influenced the abundance of males captured (*χ*^2^ = 4.4, *df* 1, *P* < 0.05, *n* = 24) with males caught at higher rates when the traps were placed on the left hand side of the cage.

#### BG-Lure Inside MAST Capture Chamber

There was a significant increase (*χ*^2^ = 13.7, *df* = 1, *P* < 0.05, *n* = 21) in the mean abundance of male *Ae. aegypti* caught by MAST Spray traps with the BG-Lure placed inside the capture chamber (11. 1 ± 2.8) compared to MAST Spray traps without a BG-Lure (7. 5 ± 1.9; Fig. 1B). Trap location had no significant influence on the abundance of males caught (*χ*^2^ = 0.2, *df* = 1, *P* = 0.7, *n* = 21).

Lastly, there was a significant increase (*χ*^2^ = 10.5, *df* = 1, *P* < 0.05, *n* = 24) in the mean abundance of male *Ae. aegypti* caught between MAST Sticky versions with the BG-Lure placed inside the capture chamber compared to those without. The MAST Sticky caught a mean per trial of 16. 8 ± 2.5 male *Ae. aegypti* whereas the MAST Sticky with the BG-Lure placed inside the capture chamber caught a mean per trial of 21. 0 ± 4.2 males (Fig. 1C). Lastly, there was no significant influence on the abundance of males caught by the trap location (*χ*^2^ = 0.6, *df* = 1, *P* = 0.4, *n* = 24).

### Field Trials

#### Total Catches

Of the total 4,644 invertebrates sampled in Cairns by all 4 trap types, 1,123 male and 764 female *Ae. aegypti* were sampled (Table 1). The only other mosquito species in which more than 10 individuals were captured was *Culex quinquefasciatus* (Say, Diptera: Culicidae), with 183 males and 180 females sampled. The most common non-mosquito bycatch was in the order Diptera (1,687 sampled), with 692 other invertebrates captured.

**Table 1.** Summary data of all taxa caught in Cairns, Australia and Madang, PNG

	Cairns, Australia				Madang, PNG			
**Taxa**	**BGS Trap**	**BGS trap + BG-Lure**	**MAST**	**MAST + BG-Lure**	**BGS trap**	**BGS trap** **+ BG-Lure**	**MAST**	**MAST + BG-Lure**
*Aedes aegypti* male	254	400	285	184	27	23	43	14
*Aedes aegypti* female	292	471	1	0	67	46	0	0
*Aedes albopictus* male	0	0	0	0	44	102	172	115
*Aedes albopictus* female	0	0	0	0	250	300	0	0
*Aedes notoscriptus* (Skuse, Diptera: Culicidae) female	6	2	0	0	0	0	0	0
*Culex annulirostris (Skuse, Diptera: Culicidae)*	0	0	0	0	1	1	0	0
*Culex quinquefasciatus* male	69	98	9	7	1,411	1,158	2	2
*Culex quinquefasciatus* female	91	89	0	0	1,025	926	0	0
*Mansonia* sp. Female	0	0	0	0	1	9	0	0
*Toxorhynchites* sp. female	3	4	0	0	0	0	0	0
Diptera (other)	774	908	0	5	4,043	5,195	9	49
Hemiptera	1	4	0	0	740	606	4	0
Lepidoptera	270	271	0	0	393	266	2	1
Hymenoptera (winged)	19	28	0	1	158	214	4	0
Formicidae	7	13	0	1	84	197	7	1
Coleoptera	26	17	0	0	121	138	0	2
Collembola	2	2	0	0	96	93	0	1
Araneae	17	11	0	0	27	24	1	0
Blattodea	1	1	0	0	25	23	0	0
Orthoptera	0	0	0	0	2	0	0	0
**Total**	**1,832**	**2,319**	**295**	**198**	**8,515**	**9,321**	**244**	**185**

From Madang, 18,265 invertebrates were sampled in total including 433 male and 550 female *Ae. albopictus* (Table 1). Few *Ae. aegypti* were sampled (107 males and 113 females). *Cx. quinquefasciatus* mosquitoes were again commonly captured (2,573 males and 1,951 females). The most common non-mosquito bycatch were also from the order Diptera (9,296 sampled), with 3,230 other, non-dipteran, invertebrates remaining.

#### Male *Aedes* Catches per Trap Type

**Fig. 2.**
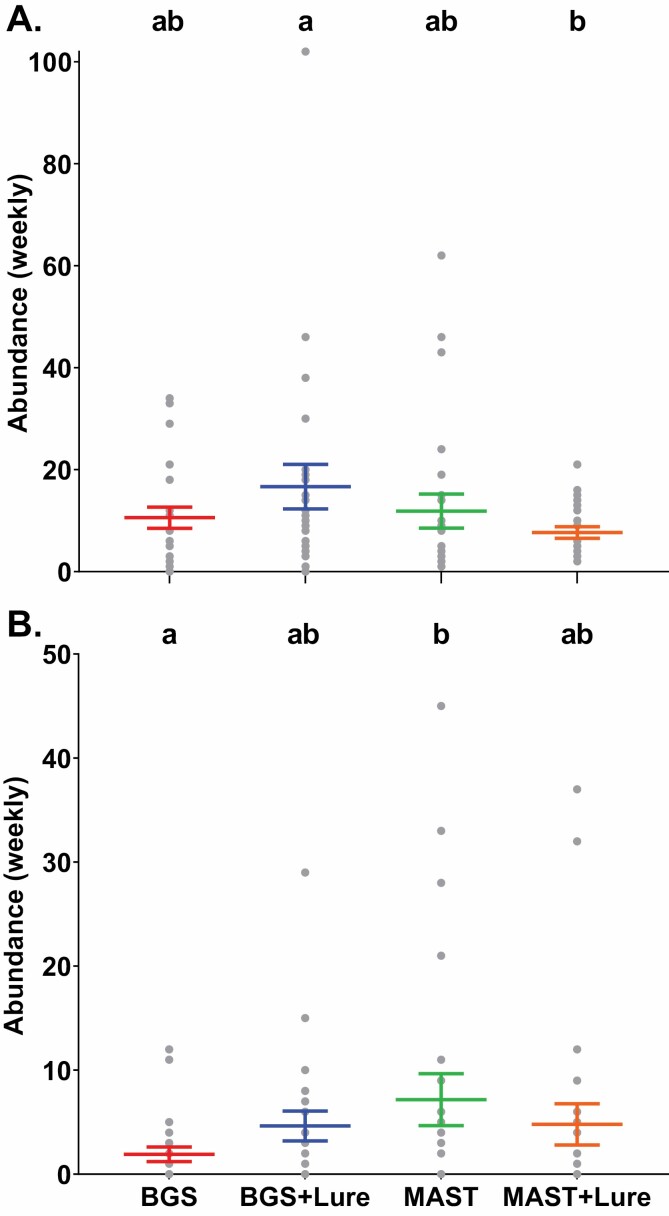
Weekly catch rates of (A) male *Ae. aegypti* in Cairns and (B) male *Ae. albopictus* in Madang. Different letters above points indicate significantly different groups (Tukey HSD, *P* < 0.05; *n* = 24). MAST captures in Cairns and Madang were made using MAST Spray and MAST Sticky trap versions, respectively.

Mean weekly abundance of male *Ae. aegypti* caught significantly differed (*χ*^2^ = 8.3, *df* = 3, *P* < 0.05, *n* = 24) between trap types in Cairns (Fig. 2A). Catches of male *Ae. aegypti* decreased, although not significantly, in MAST Spray traps with BG-Lures (7. 7 ± 1.2) compared to MAST Spray traps without (11. 9 ± 3.3; Fig. 2A). However, catches of mean (± S. E.) male *Ae. aegypti* increased, although not significantly, in BGS traps with BG-Lures (16. 7 ± 4.4), compared to those without (10. 6 ± 2.1; Fig. 2A). BGS traps with BG-Lures caught significantly more male *Ae. aegypti* than MAST Spray traps with BG-Lures and there were no significant difference in weekly mean male *Ae. aegypti* catch rates between unbaited BGS traps and MAST Spray traps (Tukey HSD, *P* < 0.05; Fig. 2A).

Mean weekly abundances of male *Ae. albopictus* caught in Madang significantly differed (*χ*^2^ = 9.0, *df* = 3, *P* < 0.05, *n* = 24) between trap types (Fig. 2B). Male *Ae. albopictus* mean weekly catches decreased, although not significantly, in MAST Sticky traps with BG-Lures (4. 8 ± 2) compared to MAST Sticky traps without (7. 2 ± 2.5; Fig. 2B). However, catches of mean (± S. E.) male *Ae. albopictus* increased, although not significantly, in BGS traps with BG-Lures (4. 6 ± 1.4), compared to those without (1. 9 ± 0.7; Fig. 2B). Unbaited BGS traps caught significantly less male *Ae. albopictus* than MAST Sticky traps without BG-Lures and weekly mean male *Ae. albopictus* catch rates did not significantly differ between baited BGS traps and MAST Sticky traps with BG-Lures (Tukey HSD, *P* < 0.05; Fig. 2B).

#### BG-Lure Influence on BGS Catches of Other Taxa of Interest

Mean female *Ae. aegypti* catch rates in Cairns were significantly greater in BGS traps containing BG-Lures, compared to BGS traps without BG-Lures (*P* = 0.008; Table 2). In Madang, mean female *Ae. albopictus* catch rates were higher in the BGS traps with BG-Lures, but the difference was not statistically significant (*P* > 0.05; Table 2). The addition of the BG-Lure to the BGS trap also did not significantly affect the catch rates of male or female *Cx. quinquefasciatus*, non-culicid dipterans or other arthropods in both locations. Although a marginally significant difference was noted with more non-culicid dipterans caught in BGS traps baited with BG-Lures than those without (*P* = 0.06; Table 2).

**Table 2.** BG-Lure influence on catches of other taxa of interest (analysis of variance)

	Taxa	BGS trap mean ± (S.E.)	BGS trap + BG-Lure mean ± (S.E.)	χ^2^	df	*P* value
Cairns	***Ae. aegypti* (female)**	**12.2 (2.1)**	**19.6 (5.4)**	**6.80**	**1**	**0.01**
	*Cx. quinquefasciatus* (male)	2.8 (1.2)	4.1 (1.5)	0.08	1	0.77
	*Cx. quinquefasciatus* (female)	3.8 (1.5)	3.7 (1.6)	1.28	1	0.26
	Diptera (non-mosquito)	32.3 (10.4)	37.8 (7.3)	0.79	1	0.37
	All non-dipteran bycatch	14.2 (2.7)	14.3 (1.3)	0.30	1	0.59
Madang	*Ae. albopictus* (female)	10.9 (2.2)	13.6 (5.4)	1.55	1	0.21
	*Cx. quinquefasciatus* (male)	61.4 (22.6)	52.6 (14.5)	0.14	1	0.71
	*Cx. quinquefasciatus* (female)	44.6 (11.3)	42.1 (11.7)	0.02	1	0.89
	Diptera (non-mosquito)	175.8 (22.6)	236.1 (36.2)	3.51	1	0.06
	All non-dipteran bycatch	71.5 (8.9)	71 (11.0)	0.73	1	0.39

The group determined to be significantly different (*P* ≤ 0.05, *n* = 24) is in boldface type.

## Discussion

Despite promising semi-field results, the addition of the BG-Lure inside the MAST capture chamber did not significantly change catch rates and, if anything, may have even repelled male *Ae. aegypti* and *Ae. albopictus* in the field. This finding is inconsistent with the positive influences of the human skin mimic lures on male *Aedes* catch rates in BGS traps in previous work (Pombi et al. 2014, Roiz et al. 2016, Amos et al. 2020, Visser et al. 2020). The BG-Lure was developed specifically for use with the BGS trap. Potentially, placing the lure on top of the MAST may have drawn male activity away from the MAST entrance so these mosquitoes were less likely to respond to the sound lure (Martin Geier, personal communication, Biogents). Additionally, if the concentration of the BG-Lure is too high it may repel, rather than attract, mosquitoes (Martin Geier, personal communication, Biogents). It is therefore possible that the concentration of olfactory cues from the BG-Lure inside the capture container increased over time to a degree that was repellent rather than attractive. If so, this may explain why this lure was attractive to mosquitoes during the short semi-field trials, but repellent over weekly field trials. Alternatively, a range of additional factors, including female behavior, may influence differences between semi-field and field results. Future field trials should investigate the influence of different concentrations and/or types of olfactory lures in the MAST system as well their placement, relative to the capture chamber of the MAST (e.g., in the MAST base or on a trap extension, rather than capture chamber). Additionally, future semi-field trials could consider running experiments over longer time-periods, whereby captured males are replenished by introducing an equal number of new males, to potentially better reflect traps captures in field conditions.

Unlike MAST traps, the addition of the BG-Lure to the BGS trap significantly increased captures of female *Ae. aegypti* in Cairns and positively, though not significantly, influenced trap catches of male *Ae. aegypti* in Cairns and both male and female *Ae. albopictus* in Madang. These results are consistent with other trials, which found that the deployment of BGS traps with BG-Lures increased catch rates of *Ae. aegypti* and *Ae. albopictus* (Barrera et al. 2013, Pombi et al. 2014, Arimoto et al. 2015, Visser et al. 2020). Our findings contradict work performed in northern Queensland where BGS traps deployed with human skin scent lures did not significantly increase female *Ae. aegypti* catch rates (Williams et al. 2006). Williams et al. (2006) suggested that mosquito strains of different provenance may be unequally attracted to olfactory lures. While *Ae. aegypti* in Cairns now harbour *Wolbachia*, this has not been demonstrated to alter the attraction of *Ae. aegypti* to human odors (Turley et al. 2014, Lau et al. 2020). Potentially, changes in both the BGS trap and BG-Lure from versions deployed in the work by Williams et al. (2006) 15 yr ago (Akaratovic et al. 2017) may also have impacted the differences seen.

While MASTs without BG-Lures caught similar numbers of male *Ae. aegypti* to unbaited BGS traps and less males than baited BGS traps, they caught more male *Ae. albopictus* than both baited and unbaited BGS traps. These results support previous *Ae. aegypti* trials in the Pacific region and Central America where MASTs captured comparable mean abundances of male *Ae. aegypti* and/or *Ae. albopictus* to unbaited BGS traps, but without the high numbers of associated bycatch (Staunton et al. 2021a, Staunton et al. 2021b). The high sensitivity of the MAST Sticky for male *Ae. albopictus* in PNG is very encouraging, given the distribution of insecticide resistance found in *Aedes* (Rivero et al. 2010) which would limit the use of traps requiring insecticides in surveillance systems.

Lastly, the addition of BG-Lures had little impact on the bycatch sampled by BGS traps. BGS traps did not catch significantly higher mean abundances of male or female *Cx. quinquefasciatus*, non-culicid dipterans or other invertebrates in Cairns or Madang when they were deployed with BG-Lures. Consequently, for *Aedes*-specific programs, the time spent sorting through bycatch from BGS traps is not likely to be greatly enhanced when these traps are deployed with BG-Lures, which is a positive operational consideration. Additionally, it is worth noting that these results do not support the integration of BG-Lures with BGS traps to enhance collections of *Cx. quinquefasciatus* in these locations, which is consistent with findings from other work performed in China (Xie et al. 2019).

### Conclusion

The positive influence of BG-Lures on BGS trap catches of *Ae. aegypti* and *Ae. albopictus* was consistent with previous trials but different to the only previous study in northern Queensland. While further development is required to successfully integrate a chemical lure within the MAST system, these trials demonstrate the high sensitivity of the unbaited MAST relative to baited BGS traps, especially for male *Ae. albopictus* in Madang. Additionally, these results further support previous findings of very limited bycatch being captured in MAST traps. Lastly, the effort to sort through bycatch associated with BGS traps, including *Cx. quinquefasciatus*, is unlikely to greatly increase with the additional deployment of BG-Lures in this surveillance trapping system. This study provides useful information for the surveillance of *Ae. aegypti* and *Ae. albopictus*, but also contributes to the further development of an effective sound trap system.

## Supplementary Material

tjab121_suppl_Supplementary_Material
