# Supplementary material for: Effect of BG-Lures on the Male Aedes (Diptera: Culicidae) Sound Trap Capture Rates
Source: J Med Entomol. 2021 Jul 8;58(6):2425–31. doi: 10.1093/jme/tjab121 (PMC8577766; doi:10.1093/jme/tjab121)

**Supplementary information**

**Figure S1.** The addition of the BG-Lure either a) on top of or b) inside of, the MAST capture chamber.


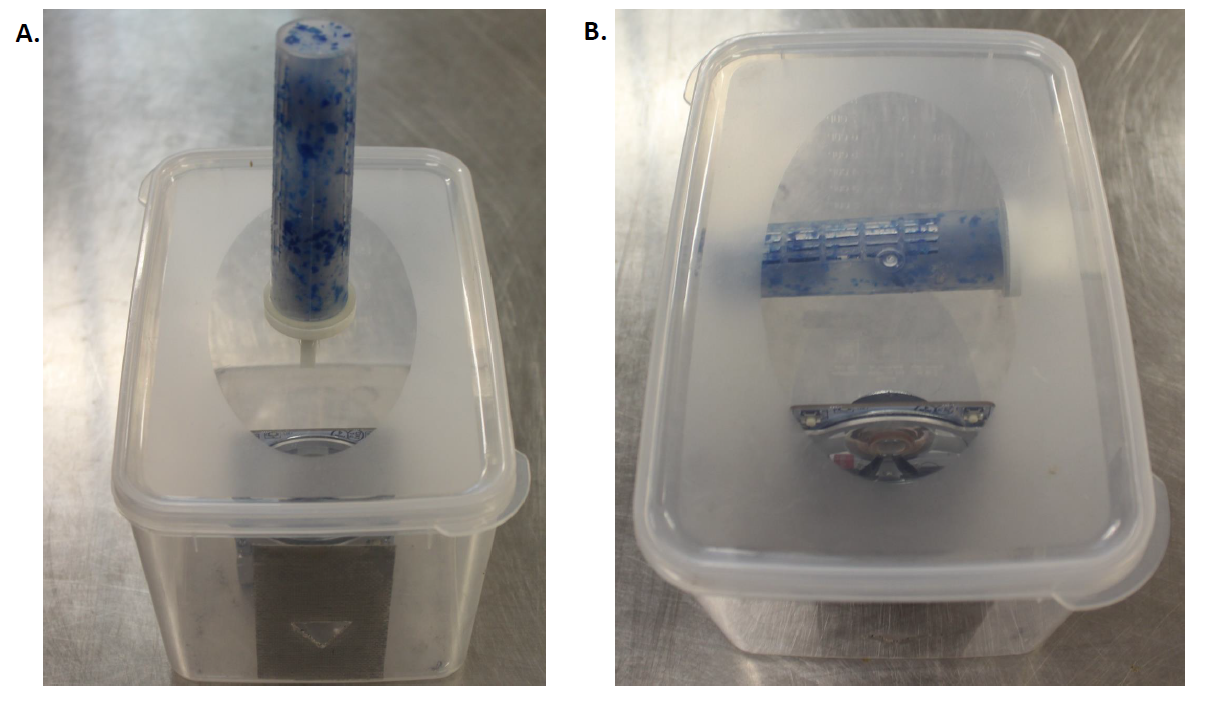

Supplement: tjab121_suppl_Supplementary_Material [file tjab121_suppl_supplementary_material.docx]
